# An unexpected phosphate binding site in Glyceraldehyde 3-Phosphate Dehydrogenase: Crystal structures of apo, holo and ternary complex of *Cryptosporidium parvum *enzyme

**DOI:** 10.1186/1472-6807-9-9

**Published:** 2009-02-25

**Authors:** William J Cook, Olga Senkovich, Debasish Chattopadhyay

**Affiliations:** 1 Department of Pathology, University of Alabama at Birmingham, Birmingham, AL 35294, USA; 2 Center for Biophysical Sciences and Engineering, University of Alabama at Birmingham, Birmingham, AL 35294, USA; 3 Department of Medicine, University of Alabama at Birmingham, Birmingham, AL 35294, USA

## Abstract

**Background:**

The structure, function and reaction mechanism of glyceraldehyde 3-phosphate dehydrogenase (GAPDH) have been extensively studied. Based on these studies, three anion binding sites have been identified, one 'Ps' site (for binding the C-3 phosphate of the substrate) and two sites, 'Pi' and 'new Pi', for inorganic phosphate. According to the original flip-flop model, the substrate phosphate group switches from the 'Pi' to the 'Ps' site during the multistep reaction. In light of the discovery of the 'new Pi' site, a modified flip-flop mechanism, in which the C-3 phosphate of the substrate binds to the 'new Pi' site and flips to the 'Ps' site before the hydride transfer, was proposed. An alternative model based on a number of structures of *B. stearothermophilus *GAPDH ternary complexes (non-covalent and thioacyl intermediate) proposes that in the ternary Michaelis complex the C-3 phosphate binds to the 'Ps' site and flips from the 'Ps' to the 'new Pi' site during or after the redox step.

**Results:**

We determined the crystal structure of *Cryptosporidium parvum *GAPDH in the apo and holo (enzyme + NAD) state and the structure of the ternary enzyme-cofactor-substrate complex using an active site mutant enzyme. The *C. parvum *GAPDH complex was prepared by pre-incubating the enzyme with substrate and cofactor, thereby allowing free movement of the protein structure and substrate molecules during their initial encounter. Sulfate and phosphate ions were excluded from purification and crystallization steps. The quality of the electron density map at 2Å resolution allowed unambiguous positioning of the substrate. In three subunits of the homotetramer the C-3 phosphate group of the non-covalently bound substrate is in the 'new Pi' site. A concomitant movement of the phosphate binding loop is observed in these three subunits. In the fourth subunit the C-3 phosphate occupies an unexpected site not seen before and the phosphate binding loop remains in the substrate-free conformation. Orientation of the substrate with respect to the active site histidine and serine (in the mutant enzyme) also varies in different subunits.

**Conclusion:**

The structures of the *C. parvum *GAPDH ternary complex and other GAPDH complexes demonstrate the plasticity of the substrate binding site. We propose that the active site of GAPDH can accommodate the substrate in multiple conformations at multiple locations during the initial encounter. However, the C-3 phosphate group clearly prefers the 'new Pi' site for initial binding in the active site.

## Background

Protozoan parasite *Cryptosporidium parvum *is a common cause of water-borne diseases throughout the world [1]. There is no effective anti-cryptosporidial therapy [2,3]. Cryptosporidium infection can be lethal in immunocompromised individuals, and therefore it poses a particularly serious problem in patients with AIDS [4]. Despite the urgent need for identification of novel drug targets, very little is known about the metabolic enzymes of this relatively newly recognized human pathogen. The parasite apparently relies mainly on the anaerobic oxidation of glucose for energy production [5-8]. Therefore, glycolytic enzymes are of considerable interest as possible targets for anti-cryptosporidial drugs. One glycolytic enzyme, the glyceraldehyde 3-phosphate dehydrogenase (GAPDH), has been considered a candidate for inhibition of protozoan parasites [9,10]. By exploiting the differences in the structure of the parasitic and human GAPDH, specific inhibitors of trypanosomatid GAPDH have been designed [11]. However, the Cryptosporidium enzyme has not been characterized previously.

GAPDH plays an essential role in glycolysis by catalyzing the reversible two step oxidative phosphorylation of D-glyceraldehyde 3-phosphate (D-G3H) into 1,3-diphosphoglycerate using NAD (or NADP) as a cofactor [12]. In the first step D-G3H is covalently attached to the active site cysteine residue via nucleophilic attack on the carbonyl group of D-G3H, resulting in the formation of a thiohemiacetal intermediate. This is followed by a hydride transfer from the thiohemiacetal to NAD, leading to the formation of a thioacyl enzyme. Finally, the resulting thioester is phosphorylated through the nucleophilic attack of an inorganic phosphate ion (Pi) on the carbonyl carbon atom of the thioacyl group, which leads to the formation of 1,3-diphosphoglycerate.

The three-dimensional structures of GAPDH from a number of mammalian, bacterial and parasitic species have been determined [13-20]. GAPDHs exist as a homotetrameric protein. Based on the location of sulfate ions originating from the crystallization medium, two anion binding sites were initially identified in each GAPDH subunit [19,21]. These sites are labeled 'Ps', corresponding to the binding site for the C-3 phosphate of D-G3H, and 'Pi', corresponding to the binding site for the inorganic phosphate. The 'Ps' site is conserved in various GAPDH structures. However, two possible 'Pi' sites have been proposed [17,19]. The original Pi site was identified in crystals grown from ammonium sulfate solutions and was based on the position of the sulfate ion [19]. The second 'Pi' site was identified by Kim et al [17] in crystals grown from a phosphate-buffered medium. This latter site, referred to as the 'new Pi' site, is 2.9 Å from the original 'Pi' site.

Skarżyñski et al [19] proposed that the 'Pi' site is the location of the inorganic phosphate in the phosphorylation step. According to their 'flip-flop' hypothesis, the C-3 phosphate of the substrate binds first to the 'Pi' site in the acylation step and then flips from the 'Pi' to the 'Ps' site during the phosphorylation step. This mechanism is supported by kinetic studies [22] as well as structural studies of the *E. coli *GAPDH in which the substrate was covalently linked to the enzyme in a hemiacetal form [16] and there was no cofactor. The crystal structure of *Trypanosoma cruzi *GAPDH covalently bound to a substrate analogue also provided strong support for this model [14]. In the *E. coli *and *T. cruzi *enzyme structures, the 'Pi' site was localized to the 'new Pi' site.

However, in the *Bacillus stearothermophilus *GAPDH (BsGAPDH) ternary complex with NAD and D-G3H, the C-3 phosphate group of the non-covalently bound substrate was located in the 'Ps' site, and Didierjean et al [15] proposed that this structure represented the productive Michaelis complex. The substrate was found in the same conformation in two ternary complexes that were prepared by using different active site mutant (Cys→Ala and Cys→Ser) forms of BsGAPDH. In a recently reported crystal structure of the thioacyl intermediate of wild type BsGAPDH the C-3 phosphate is found (with partial occupancy) in the 'new Pi' site [23] in each subunit.

We have determined the crystal structure of *C. parvum *GAPDH (CpGAPDH) in a substrate free state (apo), in the NAD-bound state (holo) and in a ternary complex with NAD and D-G3H. At no stage during purification and crystallization was the protein exposed to sulfate or phosphate ions. As a result there is no sulfate or phosphate ion in any of the CpGAPDH structures. In order to form the ternary complex with the physiological substrate, the nucleophilic cysteine residue of the active site (C153), was substituted with serine following the strategy employed by Didierjean et al [15] to form the ternary complex of BsGAPDH. However, in contrast to their results, in three subunits of the CpGAPDH tetramer the C-3 phosphate is located at the 'new Pi' site and not in the 'Ps' site. In the remaining subunit, an unexpected new binding site for this phosphate is revealed. According to the accepted mechanism of enzymatic action the conformation of the substrate in two subunits (C and D) appears to be unproductive.

## Methods

### Expression and purification

We recently reported the expression and purification of wild type CpGAPDH [24]. Briefly, recombinant CpGAPDH containing an N-terminal hexa-histidine tag was expressed in *E. coli *via induction with 0.4 mM isopropyl thio-β-D-galactoside and growing the culture at room temperature for 18 hrs post-induction. The protein was purified from the bacterial extract using metal affinity chromatography (Ni-NTA, Qiagen), cation-exchange chromatography (SP Sepharose, Amersham) and size exclusion chromatography (Superdex 200, Amersham). The point mutation (C153 to S) was introduced into the recombinant plasmid using QuikChange Site-Directed Mutagenesis kit (Stratagene) according to the manufacturer's instructions. The C153S mutant CpGAPDH was expressed and purified by the same protocol as the wild type protein.

### Enzyme Activity assay

Enzyme activity assay for wild type CpGAPDH was carried out spectrophotometrically by following the increase in absorption at λ = 340 nm due to NADH formation. The reaction mixture contained 1 ml of buffer (50 mM Triethanolamine, 50 mM Na_2_HPO_4_, 0.2 mM EDTA, pH 8.8) and varying concentrations of NAD (10 – 150 μM) and D-G3H (1.47 – 5.88 mM). DL-G3H (Sigma) contained a mixture of D- and L-G3H with 50% of the D-isomers. The reaction was initiated by adding 0.8 μg of purified CpGAPDH. All measurements were repeated at least three times. Maximal reaction velocity and Michaelis-Menten constants, V_max _and K_m _values, for D-G3H and NAD were determined from Lineweaver-Burk plots by varying the concentration of either the substrate or cofactor while the other was kept constant (2.94 mM D-G3H and 150 μM NAD).

### Crystallization and data collection

Prior to crystallization, purified proteins (both wild type and the mutant) were concentrated to a final concentration of 13 mg/ml. No attempt was made to cleave the N-terminal hexa-histidine tag.

#### Apo-CpGAPDH

Crystals of wild type apo-CpGAPDH were grown at room temperature by hanging drop vapor diffusion method using 16–20% PEG 3000 and 0.2 M NaCl in 0.1 M HEPES, pH 7.5. Crystals suitable for X-ray diffraction analysis were reproducibly grown by microseeding technique using serial dilutions of crushed crystals. X-ray diffraction data were collected at the synchrotron beam line SBC-19BM at the Advanced Photon Source (APS, Argonne National Laboratory) on a MAR CCD imaging plate. Oscillation data were collected at -170°C at a crystal to detector distance of 200 mm with 1° oscillation/frame using an X-ray wavelength of 0.9998 Å. For cryo-cooling, crystals were transferred through a series of cryo-preservative solutions containing 5, 10, 15, 20 and 25% glycerol in mother liquor (1 min in each). These crystals belong to the monoclinic space group P2_1 _with unit cell dimensions of *a *= 69.36 Å, *b *= 121.65 Å, *c *= 80.00 Å and β = 92.31°. There are four CpGAPDH subunits per asymmetric unit (one tetramer), and the value of V_m _[25] is 2.33 Å^3^/Da, which corresponds to a solvent volume fraction of 47%.

#### Holo-CpGAPDH

To prepare the holoenzyme (binary complex), wild type CpGAPDH was incubated with 2 mM NAD for 3 hr on ice and then crystallized using the same method as for the apoenzyme. These crystals were isomorphous with the apo-CpGAPDH crystals. Diffraction data for these crystals were collected at SBC-19BM beam line on MAR CCD plate.

#### Ternary complex (CpGAPDH: NAD: D-G3H)

Crystals of the ternary complex were grown using the C153S mutant protein under the same conditions described for the apoenzyme. Prior to crystallization mutant protein was incubated with 2 mM NAD and 11 mM DL-G3H. X-ray diffraction data were collected at synchrotron beam line SERCAT-22BM (APS, Argonne National Laboratory). These crystals were also isomorphous with the other two forms. Data collection statistics are summarized in Table 1.

**Table 1:** Data collection and refinement statistics.

	**Apoenzyme**	**Holoenzyme**	**Ternary complex**
**Crystal data**			
a (Å)	69.36	69.12	68.00
b (Å)	121.65	121.19	120.10
c (Å)	80.00	80.33	79.28
β (°)	92.31	92.21	92.08
Vm (Å^3^/Da)	2.33	2.28	2.19
Solvent content (%)	47	46	44
			
**Data collection**			
Resolution range (Å)	50.0 - 2.2	50.0 - 2.0	20.0 - 2.0
High resolution shell (Å)	2.28–2.2	2.07 - 2.0	2.07-2.0
Reflections	68,051	87,612	84,846
Completeness (%)	98.8 (97.5)*	98.3 (90.4)	99.7 (99.3)
Rsym	0.071 (0.182)	0.068 (0.365)	0.087 (0.309)
Overall I/σ	9.6	8.9	11.9
			
**Refinement**			
R value	0.203 (0.221)	0.205 (0.240)	0.178 (0.196)
Free R value	0.245 (0.273)	0.238 (0.266)	0.210 (0.254)
Number of protein atoms	9728	10084	10077
Number of hetero atoms	0	176	230
Number of water molecules	405	419	723
Estimated coordinate error	0.25	0.23	0.20
Ramachandran plot (%) (core, allowed, generously allowed, disallowed)	87.6, 11.7, 0.3, 0.4	87.8, 11.6, 0.3, 0.3	89.9, 9.7, 0.1, 0.3
			
**Deviations from ideality**			
Bond lengths (Å)	0.006	0.006	0.006
Bond angles (°)	1.3	1.3	1.0
Dihedral angles (°)	24.3	23.8	26.8
Improper angles (°)	0.71	0.74	2.41
			
**B-factors (Å^2^)**			
All atoms	34.3	32.3	21.9
Protein atoms	34.2	32.1	21.4
NAD	N/A	37.9	16.6
D-G3H	N/A	N/A	36.4
Water molecules	36.8	34.5	29.7

* Values in parentheses refer to the outermost resolution shell.

R_sym _= ΣΣ|*I *- *I*_ι_|/Σ*I*, where *I *is the mean intensity of the N reflections with intensities *I*_i _and common indices *h*,*k*,*l*.

### Structure determination and refinement

The structure of the apo-CpGAPDH was solved by molecular replacement, using the entire *Trypanosoma cruzi *glycosomal GAPDH tetramer (1K3T) as the search model [20]. This model gave a clear solution in the rotation and translation functions, and the electron density maps were of sufficient quality to allow replacement of non-identical residues and rebuilding of several regions where there were deletions compared to the *T. cruzi *structure. Residues 185–197 could not be modeled due to very weak electron density in this area. The graphics program COOT was used for model-building [26].

Since crystals of the holoenzyme and the ternary complex (the C153S mutant) were isomorphous with those of the apo-CpGAPDH, the latter structure was simply placed into the cell for each structure and initially refined by rigid body techniques. The electron density for the loop containing residues 185–197 was clear in both of these structures. Difference electron density maps clearly showed the positions of the NAD molecules in both structures as well as the positions of the D-G3H molecules in the ternary complex (see later).

Refinement of the apo and holoenzyme structures was performed by simulated annealing using CNS [27] with the stereochemical parameter files defined by Engh and Huber [28]. No sigma cutoff was applied to the data. Five percent of the data were randomly selected and removed prior to refinement for analysis of the free R factor. The four subunits were restrained by the non-crystallographic symmetry during most of the refinement. The restraints were gradually relaxed as refinement proceeded and dropped completely at the final stages of refinement. The progress of the refinements was guided by the decrease in both the conventional and free R factors. Individual B-factors were included in the final refinements for each structure. In case of the ternary complex, after preliminary refinement of the polypeptide chains, residues S153 and H180 were mutated to glycine, and difference electron density maps calculated at this stage allowed us to place the side chains for these residues as well as the cofactor and the substrate unambiguously. Attempts to refine other orientations of the substrate resulted in short contacts and unsuccessful refinement. Refmac5 [29] was used for further refinement of the structure. Table 1 contains a summary of the refinement parameters for all three structures.

## Results

### Biochemical properties of wild type and mutant CpGAPDHs

The presence of the N-terminal hexa-histidine tag allowed application of metal affinity chromatography for rapid isolation of recombinant CpGAPDHs (both wild type and mutant) from *E. coli *extract in a nearly homogeneous form. Subsequently ion exchange and size exclusion chromatography allowed isolation of a highly purified tetrameric protein sample for enzyme kinetic analysis and crystallization. The apparent K_m _values for NAD and D-G3H for wild type CpGAPDH enzyme were estimated to be 0.032 ± 0.002 and 762.7 ± 43.0 μM, respectively, and the V_max _is 72 ± 2 μmole/min/mg. The K_m _value for NAD is in the same range as for most mammalian GAPDHs but approximately 10 fold lower than that for parasitic enzymes from *L. mexicana *and *T. brucei*. The K_m _value for D-G3H is 3 to 10 fold higher than for mammalian GAPDHs and 2 to 3 times lower than respective values for *L. mexicana *and *T. brucei *enzymes [30-33]. As observed with the BsGAPDH mutant, CpGAPDH C153S mutant possessed low but definite enzymatic activity [15]. The measured specific activity of the mutant enzyme was approximately 450 fold lower than that of the wild type enzyme.

#### Overall structure of CpGAPDH

Since the protein containing the hexa-histidine affinity tag was enzymatically active, no attempt was made to remove the tag before crystallization. Unlike the GAPDH enzymes from human placenta [13] and *Plasmodium falciparum *[34], the hexa-histidine tag did not impose any difficulty in obtaining well diffracting crystals of CpGAPDH irrespective of substrate and cofactor status.

The overall structure of CpGAPDH is very similar to other GAPDHs for which structures have been well described, so this report will focus only on important differences between CpGAPDH and other GAPDH structures. The asymmetric unit in the crystal structure contains one tetramer of CpGAPDH with subunits related by 222 non-crystallographic symmetry. Each subunit contains seven α-helices and two β-sheets, one of seven strands and another with eight. The quality of the structure in all three crystal forms (apo, holo and ternary complex) was excellent. In all three structures greater than 99% of the residues are in the allowed (core + allowed) regions of the Ramachandran plot (Table 1). Only one residue (Val243) in each subunit (in each structure) has phi/psi angles in a disallowed region; this residue occurs in a highly conserved region and assumes a similar conformation in all known GAPDH structures. The number of water molecules located in the ternary complex was considerably higher (723) as compared to those in the apo (409) and holo (418) structures, although the resolution of the structure (2.0 Å) was identical to that of the holoenzyme. The average B factor of water molecules in the ternary complex was also lower than in the other two structures (see Table 1).

#### Cofactor binding

The holoenzyme form of CpGAPDH contains one NAD molecule in each subunit. In some GAPDH holoenzyme structures NAD is found in only two or three of the four active sites [13,14,35]. This difference in the composition has been proposed to result from the cooperativity among the enzyme subunits. However, in all subunits of the CpGAPDH holoenzyme structure, NAD molecules are well ordered with an average B-factor of 38.2 Å^2 ^(for comparison, the average B-factor for the protein atoms is 32.3 Å^2^), suggesting that all four subunits are capable of binding NAD at the same time at the NAD concentration used for crystallization. It should be noted that we used 2 mM NAD for co-crystallization, in comparison to 10 mM used in the case of human placental enzyme [13], even though the K_m _for NAD is comparable for the human and CpGAPDH enzymes [32,36].

**Figure 1:**
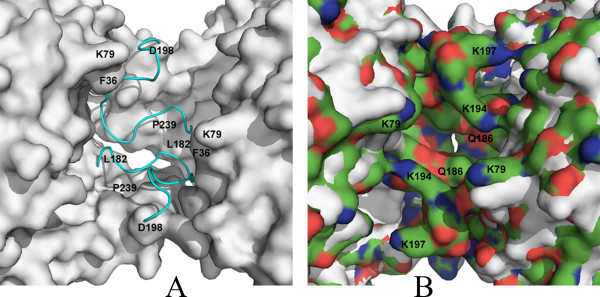
**Cofactor induced ordering of S-loop**. A: Surface drawing showing the packing of CpGAPDH subunits A and D in the apoenzyme. A loop comprising of residues 185–197 is disordered in the apoenzyme structure. CpGAPDH holoenzyme structure in which the above mentioned loop is ordered (shown in cyan), is superposed on the apoenzyme structure. B: Surface representation of the interface between subunits A and D of CpGAPDH. A and D subunits of apo-CpGAPDH and Holo-CpGAPDH superposed. The S-loop, which is disordered in the apoenzyme structure but ordered in the holoenzyme, is shown as surface colored by charge (red: negative, blue: positive, green: neutral). Interactions occur between molecules A & D and B & C.

Cofactor binding induces ordering of the S-loop (residues 185–197) in all four subunits of CpGAPDH (Fig. 1A). In the apoenzyme this loop, which forms part of the NAD binding pocket, is not visible in the electron density map apparently due to disorder in the absence of NAD. The loop is well defined in all four subunits in the holoenzyme structure as well as in the ternary complex. Ordering of this loop increases interactions between subunits positioned across from each other in the tetramer (A:D, and B:C subunits) and changes packing environment at the subunit interfaces (Fig. 1B). However, there is no direct contact between NAD and any amino acid residue in this loop. In the holoenzyme one water molecule is involved in bridging the pyrophosphate of the nicotinamide moiety with the peptide nitrogen atom of A184 in subunits A (W425), C (W717) and D (W379). The corresponding oxygen and nitrogen atoms in the B subunit are ~1Å further than in the other three subunits and no water molecule could be located for bridging them. In addition, in each subunit pair (A, D and B, C) one water molecule acts as a bridge between the main chain oxygen atom on D190 of one subunit and the adenosine pyrophosphate group of the NAD molecule belonging to the other subunit. In each subunit, one or both of the carboxyl oxygen atoms of D34 forms hydrogen bonds to the hydroxyl oxygen atoms of adenosine ribose of NAD.

**Figure 2:**
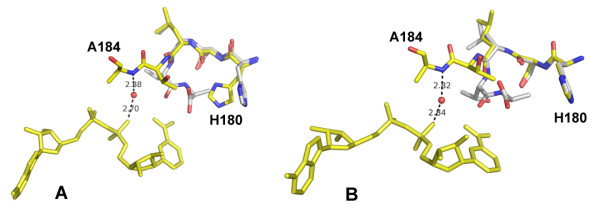
**Cofactor induced conformational changes in active site histidine (H180)**. A: Movement of the active site histidine residue in subunits A and D. Apo (white), Holo (yellow); B: In subunits B and C H180 remains in the same position in the apo and holo forms.

There is a significant shift in the main chain as well as side chain of five residues (180–184) immediately preceding the S-loop upon NAD binding. Although the movement of the polypeptide chain is similar in all subunits, this movement had varying effects on the active sites of individual subunits. In subunits A and D, the side chain of H180 moved away from its position in the apoenzyme but in B and C it remained unchanged. In the *E. coli *GAPDH, NAD binding resulted in a slight increase in the distance between the NE2 atom of the histidine and the thiol group of the cysteine residue in the active site. On the contrary, the distance between the corresponding residues in BsGAPDH decreased upon NAD binding. In CpGAPDH apoenzyme the distance between the corresponding atoms ranged between 4.55 and 4.93Å in different subunits. Upon NAD binding, the distance between the NE2 atom of H180 and SG atom of C153 decreased in all four subunits. However, in two of the subunits (A and D), these atoms moved more than 1.2Å closer (than in the apoenzyme) while in the other two subunits, the movement was relatively small (~0.3 and 0.2Å). Movement of the H180 residue in A and D subunits is shown in Fig. 2A; in B and C subunits H180 remained in the same position (Fig. 2B). Nevertheless, upon D-G3H binding the side chain of H180 in all subunits moves to a similar position.

Comparison of the structures of apoenzyme and holoenzyme reveals, in addition to ordering of the S-loop, four other regions differ significantly: residues 35–36, 77–83, 98–103 and 180–184. Residues 35–36 are in a loop that is close to the adenine ring of the NAD molecule. The movement of this loop is probably required to accommodate the NAD molecule as well as to form better hydrophobic contacts with the adenine ring of NAD. Residues 77–83 are part of a surface loop that is at one end of the NAD binding site. Movement of this loop places the main chain oxygen of K79 within hydrogen-bonding distance of AN6 on the NAD molecule. While the arrangement of this loop perhaps stabilizes the binding of NAD, movement of the loop 180–184 allows substrate binding.

In spite of slight differences in the local structure, the overall structure of the four subunits within the asymmetric unit of the binary complex is very similar. The average root-mean-square value obtained from the different pair-wise superpositions of the Cα atoms is 0.2Å. However, in the ternary complex, subunit D differs significantly from the other three subunits in the region from residues 213 to 225. The average root-mean-square value for pair-wise superpositions of the Cα atoms in subunits A, B and C is 0.25 Å, but the Cα positions of residues 213 to 225 in the D subunit differ by up to 2.9 Å from the corresponding residues in the other three subunits. In fact, this region in the D subunit assumes a conformation that corresponds to the structures of substrate-free CpGAPDH. The position of NAD is virtually identical in the binary and ternary complexes and is very similar to previously described structures.

#### Glyceraldehyde 3-phosphate binding to CpGAPDH

**Figure 3:**
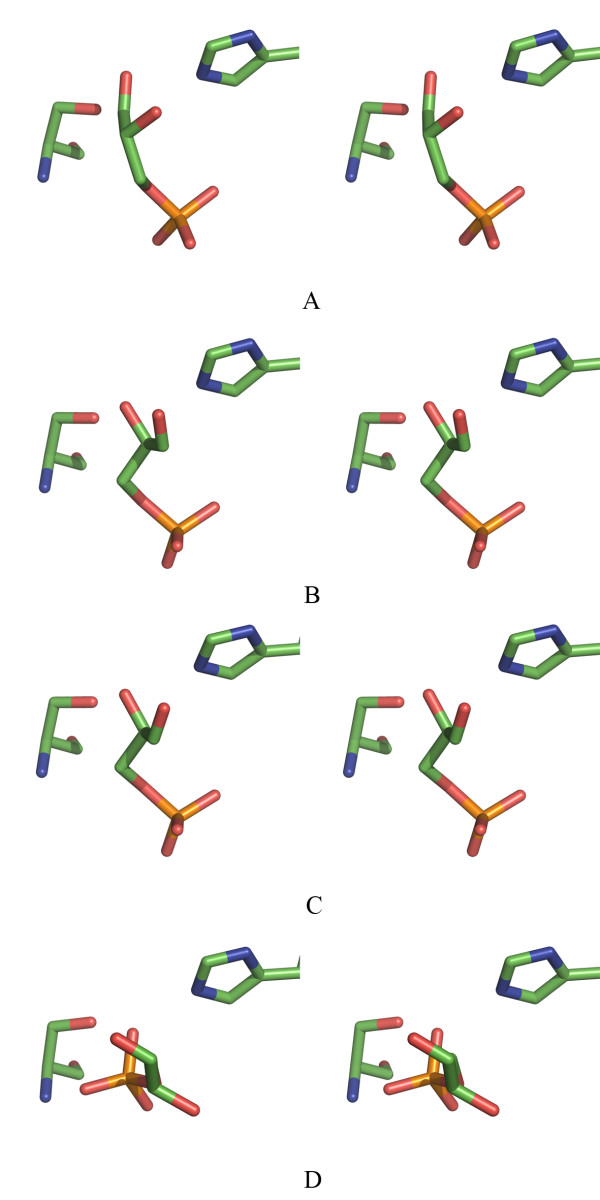
**Substrate binding in CpGAPDH subunits**. Stereo view of the substrate and active site residues (H180 and S153) shown in stick model in each subunit. A-D: Subunits A-D.

Although a racemic mixture of L- and D-G3H was used to prepare the ternary complex, only the D-enantiomer was bound to each molecule of the tetramer. An active site mutant (C153→S) was used to prepare the ternary complex. In the CpGAPDH ternary complex, substrate is bound to each subunit slightly differently than in others revealing the flexibility of the substrate binding event in the active site of GAPDH (Fig. 3). The quality of electron density for D-G3H molecule in each subunit was excellent (see Additional file 1).

**Figure 4:**
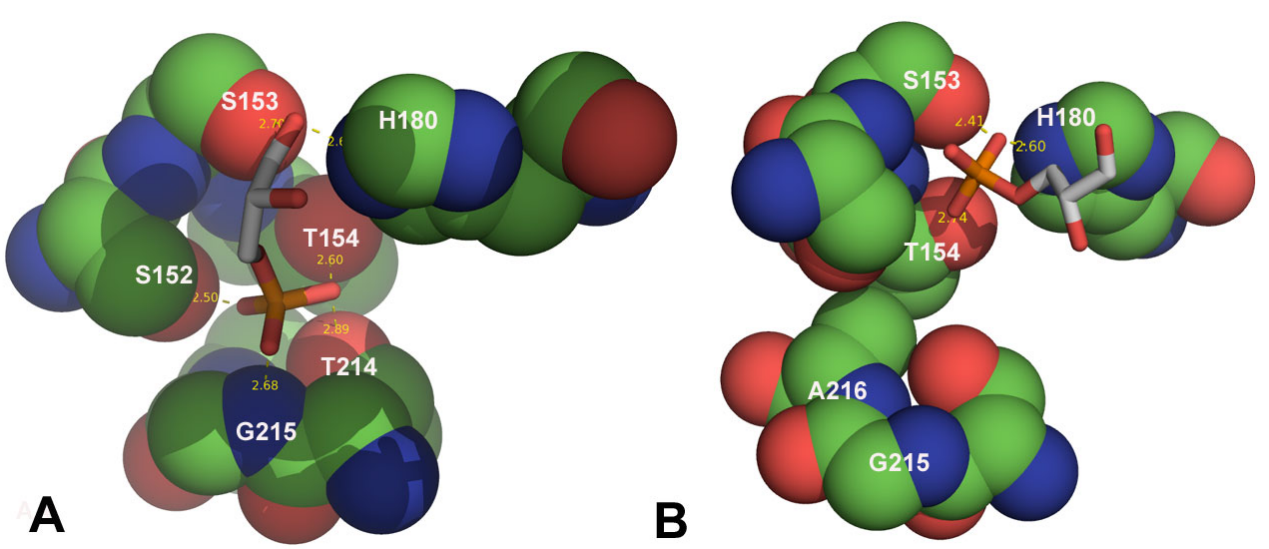
**Comparison of the D-G3H binding sites in CpGAPDH, A and D subunit**. A: Subunit A of CpGAPDH. Stick models are shown in white and red for subunit A; residues that bind to the C-3 phosphate and the active site residues (S153 and H180) are shown as sphere. The C3-phosphate occupies the 'new Pi site' in A, B and C subunits. B: Orientation of D-G3H molecule in subunit D of CpGAPDH (white and red stick) is different. There is no interaction between the phosphate oxygens and the phosphate binding loop.

Two distinct positions for the substrate have been described in different GAPDH structures (Table 2). In CpGAPDH subunits A, B and C the substrate binds with the C-3 phosphate in the classical 'new Pi site' as defined by Kim et al [17]. The phosphate oxygen atoms form hydrogen bonds with residues G215 (N), T154 (OG1), S152 (OG), A216 (N) and T214 (OG1) (Fig. 4A and Table 3). In subunit D, the phosphate moiety of D-G3H occupies a site that does not correspond to any previously reported phosphate binding site (Fig. 4B). The phosphorus atom in this subunit is ~2.6–2.8Å from the corresponding position in the other three subunits. The phosphate oxygen atoms form hydrogen bonds with residues S153 (OG), H180 (NE2), T154 (OG1) (Table 3). In subunits A, B and C the hydroxyl oxygen atom of S152 is involved in hydrogen bonding with the C3 phosphate oxygen of the substrate. As in the holoenzyme this hydroxyl oxygen also forms hydrogen bonds with the side chain hydroxyl group of T155. In subunit D, the S152 hydroxyl group retains the hydrogen bond with T155, but the phosphate is oriented away from the S152 hydroxyl oxygen.

**Table 2:** GAPDH complexes with substrate or substrate analogue.

PDB number	Reference	NAD present	Substrate	No. of subunits with substrate	Ps or New Pi* site	Soaked or Co-crystallized
CpGAPDH	This structure	Yes	D-glyceraldehyde 3-phosphate	4	New Pi^&^	Co-crystal
1GYP	*17*	Yes	PO_4_	4	Ps and new Pi	Co-crystal
1DC4	*16*	No	D-glyceraldehyde 3-phosphate	2^#^	New Pi	Soaked
1NQO	*15*	Yes	D-glyceraldehyde 3-phosphate	4	Ps	Soaked
3CMC	*12*	Yes	D-glyceraldehyde 3-phosphate	4	New Pi	Soaked
1ML3	*14*	Yes	(3-formyl-but-3-enyl)-phosphonic acid	3^+^	Ps	Co-crystal
1QXS	*18*	Yes	1,3-biphospho-D-glyceric acid	1^$^	Ps	Co-crystal

* The 'new Pi' site is defined as the site of the phosphate in 1GYP, which contains only phosphate and NAD.

^&^Only subunits A, B and C. The D-G3H molecule in subunit D occupies a different position that does not correspond to any previously reported phosphate position.

^# ^Two subunits per asymmetric unit.

^+ ^Two conformers in the structure. Subunit D has only conformer A, and its phosphate moiety binds in the 'Ps' site. Subunits B and C have both conformers at 50% occupancy; the phosphate moiety in conformer B binds in a site that is 2.5 to 2.8 Å from the 'new Pi' site.

^$ ^Only one subunit (subunit C) binds the analogue. One of the phosphate moieties binds in the 'Ps' site. The other binds in a site that is 2.8 Å from the 'new Pi' site.

**Table 3:** Interactions between D-G3H and the active site residues of C153S CpGAPDH

		Distance in Subunit			
D-G3H	Residue (atom)	A	B	C	D
O1	S153 (OG)	2.70			
O1	H180 (NE2)	2.64			
O1	R237 (NH2)			3.05	
O1	Water (O)		2.61	2.71	2.52
O2	S153 (OG)		2.70	2.66	
O2	H180 (NE2)		2.75	2.75	
O2	R237 (NH2)				3.17
O2	Water (O)	2.66			
O2P	S153 (N)				3.11
O2P	T154 (OG1)		2.65	2.70	
O2P	T214 (OG1)		2.84	2.75	
O2P	G215 (N)	2.70			
O2P	Water (O)	2.66	2.79	2.75	2.76
O2P	Water (O)	2.97			
O3P	S152 (OG)		2.68		
O3P	T154 (OG1)	2.59			
O3P	T214 (OG1)	2.91			2.74
O3P	G215 (N)		2.90		
O3P	A216 (N)		3.00		
O3P	Water (O)	2.82		2.66	
O3P	Water (O)			2.75	
O4P	S152 (OG)	2.49		2.70	
O4P	S153 (OG)				2.43
O4P	H180 (NE2)				2.60
O4P	T214 (O)			2.83	
O4P	T214 (OG1)			2.95	
O4P	Water (O)		2.65		
O4P	Water (O)		2.65		

**Figure 5:**
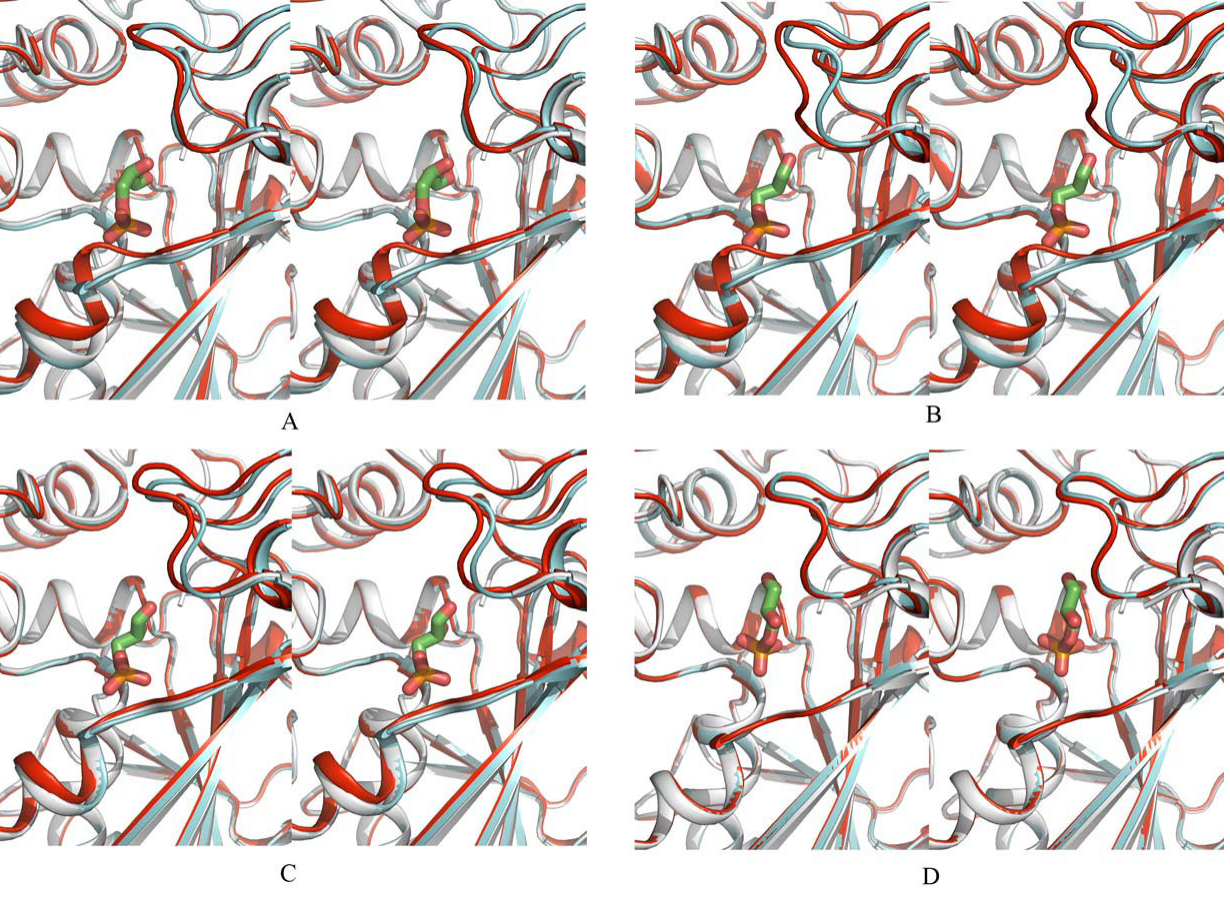
**Stereo view of the substrate induced conformational change in the phosphate binding loop**. Movement of the phosphate binding loop upon substrate binding is shown. Structure of CpGAPDH apoenzyme (white), holoenzyme (cyan) and ternary complex (red) superposed on each other and shown as cartoon for each subunit.

Upon substrate binding a significant change occurs in subunits A, B and to a lesser extent in C in the region from residues 213 to 225, which includes the last residue in a β-strand, two residues that connect the strand to a helix, and the beginning of an α-helix (Fig. 5). The conformation of this portion of the polypeptide chain appears to be determined by the presence or absence of a phosphate moiety in the 'Pi' site (see below). In the CpGAPDH ternary complex, the conformation of the polypeptide chains (in molecules A, B and C) is similar to each other and to the structures of GAPDH from *E. coli *[16] and *L. mexicana *[17], in which the phosphate groups occupy almost identical positions. However, this stretch of the polypeptide chain in subunit D of the ternary complex remains in the same conformation as in the apo and holoenzyme structures (Fig. 5D). In this subunit D-G3H molecule not only occupies a different position from that in the other three subunits, but it is oriented differently from any other reported enzyme-substrate complexes (Figs. 3 and 4).

**Figure 6:**
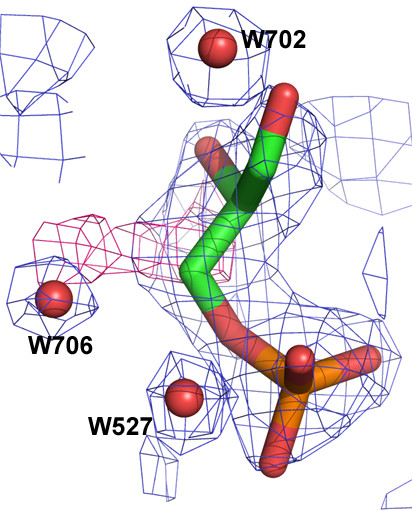
**Possible rotation or movement of the substrate in the active site**. 2Fo-Fc electron density map computed after the final cycle of refinement contoured at 1σ level (in blue) and Fo-Fc electron density map (3.0σ level) shown in pink around the substrate molecule in C subunit.

The orientation of the O2 hydroxyl group and O1 carbonyl oxygen of D-G3H with respect to the active site residues (S153 and H180) varies in different subunits of CpGAPDH tetramer. In subunit A, the O1 oxygen is within hydrogen bonding distance of NE2 of H180 as well as the hydroxyl oxygen atom of S153 (in the wild type enzyme a cysteine residue is present in its place). The O2 oxygen atom forms a hydrogen bond with a water molecule. In subunits B and C, the D-G3H is rotated approximately 180 about the C2-C3 bond (with respect to the substrate in the A subunit). In both B and C subunits, the C2 hydroxyl oxygen atom is within hydrogen bonding distance of H180 NE2 and S153 OG. In these subunits the O1 oxygen of the substrate forms a hydrogen bond with a water molecule. An Fo-Fc electron density map calculated after refinement, showed unexplained density (connecting the position of the C3 atom to a neighboring water molecule) near the D-G3H molecule in the C subunit (Fig. 6) suggesting some movement or conformational flexibility of this molecule. Attempts to fit an alternate conformation of D-G3H in this density were not successful. In molecule D, however, the substrate is rotated 90° so that one of the phosphate oxygen atoms forms a hydrogen bond with the H180 NE2 and S153 OG. There is also a hydrogen bond between the T154 side chain and a phosphate oxygen. In this subunit, the orientation of the substrate places the O1 oxygen away from the active site residues; the O2 oxygen forms a contact with side chain nitrogen atom (NH2) of R237. The position of the substrate in this subunit is clearly not suitable for catalysis and may represent an initial (Pre-Michaelis) complex. Comparison of the holoenzyme subunits with those in the ternary complex reveals that upon substrate binding the side chain of R237 residue in each subunit moves towards the substrate.

#### Comparison of substrate binding

Two other structures of a ternary GAPDH complex with NAD and D-G3H have been published (including the recently reported thioacyl intermediate of BsGAPDH), although there are several structures of complexes with a substrate analogue. There is also one structure of a binary complex that contains only the D-G3H molecule without NAD. The NAD molecule occupies a similar position in ternary complexes. However, the positions and conformations of the D-G3H molecules and analogues vary widely. Table 2 lists the most relevant GADPH structures with the substrate or substrate analog.

In the *E. coli *GAPDH structure [16], the phosphate moiety of D-G3H occupies the same position as in our structure, but the molecule is rotated along the C2-C3 bond. However, the *E. coli *GAPDH complex did not contain NAD, and the position of the carbonyl oxygen in D-G3H would not be allowed if NAD was bound in the usual position. In two ternary complexes of BsGAPDH, the phosphate was found to bind in the Ps site [15]. In forming these complexes the substrate was soaked into crystals of mutant forms of holoenzyme (active site cysteine was mutated to alanine or serine). The substrate bound non-covalently with the phosphate in the 'Ps' site; the C2 hydroxyl group pointed towards the main chain nitrogen of residue 149 (Bs numbering). Orientation of the O1 oxygen in the complex with the serine mutant is suitable for accepting hydrogen bonds from the NE2 atom of the catalytic histidine and the hydroxyl group of the serine residue. This complex was referred to as the 'Michaelis complex'. More recently the crystal structure of the thioacyl intermediate of BsGAPDH has been reported [12]. This complex was prepared by soaking D-G3H into a crystal of holoenzyme (wild type). In this structure the C-3 phosphate of the substrate occupies the 'new Pi' site and the substrate is oriented in a different manner than in the previous complex.

Crystal structures of two other ternary complexes of GAPDH containing analogues of D-G3H have been reported. In the *T. cruzi *GAPDH structure with (3-formyl-but-3-enyl)-phosphonic acid [14], three of the four subunits bind the analogue in the active site, but there are two distinct conformers of the analogue. The phosphate moiety in conformer A binds in the 'Ps' site (subunit D); in conformer B it binds in the 'Pi' site (subunits B and C). Subunit D has only conformer A; subunits B and C have both conformers at 50% occupancy. Orientation of O1 and O2 atoms in CpGAPDH subunits matches with some of the conformers in this structure. The phosphate atom in CpGAPDH (subunits A-C) is ~2.8Å from its position in the 'Pi' site in the *T. cruzi *enzyme complex. In the *T. cruzi *GAPDH structure with 1,3-biphospho-D-glyceric acid [18], only one subunit binds the analogue. One of the two phosphate moieties of the analogue occupies the 'Ps' site; the other is about 2.9Å from the 'Pi' site.

#### Comparison with human GAPDH

Alignment of the CpGAPDH sequence with those of other parasitic GAPDHs and human GAPDH shows that CpGAPDH is very similar to *Plasmodium falciparum *GAPDH [34]. Like Plasmodium GAPDH, a dipeptide (KG) insertion after residue 193 is present. Electron density for this dipeptide, which belongs to the S-loop, was excellent in all four subunits of the holoenzyme and ternary complex. Pair wise sequence comparison of human and *C. parvum *GAPDH sequences also showed three additional single residue insertions, K74, N127 and K144, in the CpGAPDH sequence. These residues are at the protein surface.

**Figure 7:**
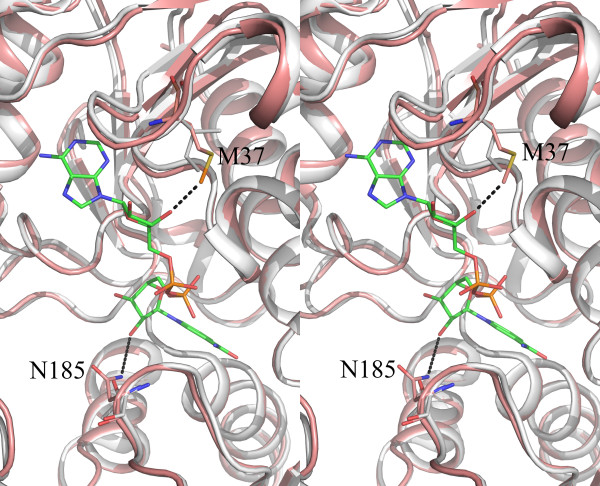
**Stereo view of the NAD binding site**. Cartoon representation of CpGAPDH (holo, subunit A) in wheat color superimposed on the O subunit of human GAPDH holoenzyme structure (PDBID: 1ZNQ) shown in white. CpGAPDH residues M37 and N185 are shown in stick (color coding C: orrange, N:blue, O: red and S: yellow) along with corresponding human GAPDH residues I38 and T184(C: white, N: blue and O: red). NAD molecule is also shown in stick (C: rose, N: blue and O: red). NAD atoms nearest to M37 and N185 are connected with dotted lines.

As noted in the Introduction section, the NAD binding pocket of trypanosomal GAPDH has been targeted for designing specific inhibitors. However, the amino acid residues near the NAD binding pocket that allowed exploitation of this site in trypanosomal GAPDH are conserved in human and *C. parvum *GAPDH. The only residues near the NAD binding pocket of CpGAPDH which are different in the human enzyme are M37 and N185, corresponding to residues I38 and T184 in human GAPDH (Fig. 7). In the holoenzyme the side chain atom CE of M37 packs closely to the hydroxyl oxygen (O3B) of the adenosine ribose in NAD (3.5 to 3.6Å in the four subunits). The CD1 atom of the corresponding residue I38 in human GAPDH is 3.4Å from this ribose oxygen. The distance from the side chain N or O of N185 to the nearest ribose oxygen atom at the nicotinamide end of NAD (OD2) varies from 5.6 to 6.8 Ǻ in different subunits of CpGAPDH. Further studies will be necessary to determine if the subtle differences in the NAD binding site of human and CpGAPDH can be exploited for developing specific inhibitors.

## Discussion

A large number of studies on bacterial and eukaryotic GAPDHs have been devoted to understanding the reaction mechanism of these enzymes [37]. These studies have been supplemented by a number of structural investigations in an effort to characterize the active site interactions with the substrate and cofactor [38]. Many of these studies have been conducted elegantly with clever tactics to capture the substrate at various stages of the reaction for which the mechanism has been fairly well established. However, as listed in Table 2, results of these structural studies showed variations in the binding mode and orientation of the substrate with respect to the active site residues revealing the plasticity of the GAPDH active site.

In order to capture a stable ternary complex of CpGAPDH with NAD and the physiological substrate we used an active site mutant that had extremely low but definite enzymatic activity. It is important to note that there is a major difference in the preparation of various complexes of GAPDH. While both BsGAPDH ternary complexes were obtained by soaking crystals of the binary complex in a solution containing DL-G3H, the CpGAPDH ternary complex was prepared by incubating the protein solution with NAD and DL-G3H for 3 hrs on ice prior to setting up crystallization. In order to maintain the phosphate binding sites unoccupied, we avoided using sulfate or phosphate ion during purification or crystallization. In preparing the BsGAPDH complexes, sulfate (as ammonium salt) was used either during purification or in crystallization. Some of the *E. coli *complexes were obtained by soaking cofactor and substrate into apo crystals.

In the non-covalent BsGAPDH/NAD/substrate complex the C-3 phosphate is bound to the 'Ps' site, and the orientation of the D-G3H molecule is appropriate for interaction of the O1 atom with the NE2 atom of active site histidine and OG of serine (substituted for cysteine). The C-3 phosphate interacts with the side chain nitrogen atom of R231, side chain oxygen atom of T231 and the 2'-hydroxyl group of ribose attached to the nicotinamide ring of NAD. The R195 side chain forms a weaker interaction (3.14Å) with O3P. In the thioacyl enzyme intermediate positions of both the phosphate group and the O1 atom change. According to Moniot et al [23] this represents the binding mode after flipping of the substrate that occurs concomitantly or after the redox step. In this structure in each subunit a sulfate ion is bound at the 'Ps' site. The substrate molecule is partially occupied in the active site with the C-3 phosphate binding to the 'new Pi' site along with a sulfate ion that also partially occupies this site. The 206–210 loop also exists in alternate conformations in forming the 'Pi' site. While the C1 atom is covalently linked to the thiol group of C149, the O2 atom forms a hydrogen bond with the nicotinamide oxygen.

The structure of the ternary complex of CpGAPDH demonstrates the extremely flexible nature of the GAPDH active site. In the A, B and C subunits, the phosphate occupies the 'new Pi' site and the movement of the phosphate binding loop is obvious. In the A subunit, there are strong interactions between the O1 carbonyl oxygen of the substrate and the active site H180 and S153 side chains as indicated by uninterrupted electron density covering this area of the subunit (see Additional file 1). In the B subunit the orientation of the O1 and O2 atoms with respect to the substrate is different with the O2 hydroxyl oxygen now directed toward the active site residues. Electron density between the substrate and the side chains of S153 and H180 is not continuous in this active site. In the C subunit the electron density indicates movement (or rotation) of the substrate (Fig. 6). However, attempts to refine multiple conformations of D-G3H in this active site were not successful. In our model, the electron density map showed interaction of the O2 atom with both NE2 of H180 and OG of S153. In the D subunit, the phosphate, on the other hand, interacts with both NE2 of H180 and OG of S153. The phosphate binding loop in this subunit remains in the substrate-free conformation (as in the apo and holoenzyme structures). Binding of the C3 phosphate in the A, B, C subunits suggest that the 'new Pi' site is preferred.

The position of the C-3 phosphate in the CpGAPDH structure (A, B and C subunits) is in agreement with several other structural studies, including the structure of the BsGAPDH in complex with the substrate analog glycidol 3-phosphate (and NAD) [19], and the structure of *E. coli *GAPDH in complex with D-G3H (and no NAD) [16]. In both structures, the C-3 phosphate group is located in the 'new Pi' site. Each subunit of CpGAPDH contains a fully occupied NAD molecule. Therefore, the preference of the C3 phosphate in the 'new Pi' site is not due to the lack of integrity (or completeness) of the 'Ps' site as was suggested to be the case for *E. coli *GAPDH.

Comparison of the structure of *T. cruzi *GAPDH in the holo form and in the apo form in complex with an inhibitor showed major structural changes were related to the orientation of residue R249 (*T. cruzi *numbering). In the absence of substrate (or analogue) or anion in the active site, the side chain of R249 points to D210. At the same time the distance between the NE2 atom of the active site histidine and the SG atom of the active site cysteine increased upon binding of the substrate analogue. In all subunits of CpGAPDH the distance between the arginine (R237) and aspartate (D198) residues increased significantly upon substrate binding. In each subunit of the ternary complex R237 points towards T183, and the contact distance between the side chain nitrogen atom of R237 and OG1 atom of T213 ranges from 2.60 to 3.19Å.

In the ternary complex the distance between H180NE2 and S153OG in different subunits varied in the range of 4.11–4.23Å. The corresponding distances in the BsGAPDH non-covalent complex were very similar to the latter distances (4.08–4.31Å) suggesting that the active site residues were perfectly positioned for the formation of the productive complex in each subunit of CpGAPDH.

## Conclusion

**Figure 8:**
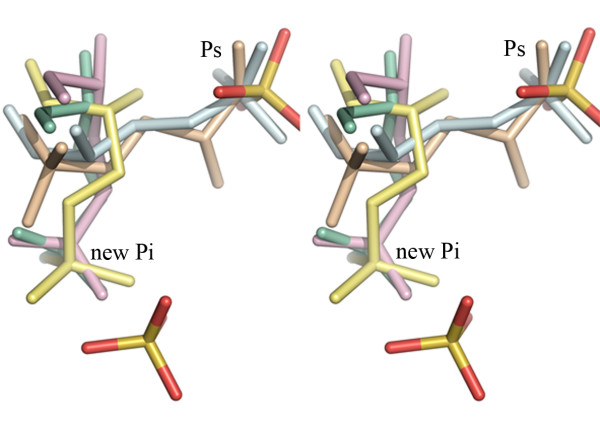
**Flexibility in substrate binding at the GAPDH active site (stereo)**. Position of D-G3H in the CpGAPDH ternary complex subunit A (yellow), subunit D (wheat); mutant BsGAPDH ternary complex (PDBId: 1nqo) subunit O (pale cyan); BsGAPDH thiacyl intermediate (PDBId: 3cmc) subunit O (light green); *E. coli *GAPDH (1dc4) subunit O (light pink). The 'Ps' and 'new Pi' sites are indicated. A sulfate ion is bound in each subunit of the BsGAPDH complex.

Structural data on GAPDHs in complex with substrate and substrate analogs demonstrate extraordinary plasticity of the active site. It is evident from these studies that the active site can accommodate the substrate in a variety of positions and conformations (Fig. 8). Direct comparisons of the available structures are not always straightforward due to the differences in the composition of the samples and conditions used for crystallization. In particular, the presence of sulfate (or phosphate) ions in the purification and crystallization media for some of these complexes changes the charge distribution and binding environment. For example, sulfate ions are located in the 'Pi' sites in the thioacyl derivative of BsGAPDH/NAD/D-G3H complex (Fig. 8). How these ions influence the binding and movement of the substrate in the active site is not clear. However, structure of the ternary complex of CpGAPDH presented here clearly shows preference of the C3-phosphate for the 'new Pi' site for binding. Moreover, the presence of multiple conformations of D-G3H in various structures and in different subunits of CpGAPDH also demonstrates the flexibility and movement of the substrate even when the C-3 phosphate is bound at the 'new Pi' site.

The atomic coordinates and structure factors for CpGAPDH structures have been deposited with the Brookhaven Protein Data Bank (PDBIds: 3CHZ, 3CIE and 3CIF).

## Authors' contributions

WJC was involved in structure solution, model building and refinement, OS was responsible for cloning, expression, purification, enzyme assay, crystallization, intensity data collection, model building and refinement. DC was responsible for overall supervision, intensity data collection, data processing, model building and refinement. All authors contributed to manuscript preparation.

## Supplementary Material

Additional file 1 Substrate binding site in CpGAPDH subunits. Fo-Fc electron density maps contoured at 3.0σ level computed before placement of D-G3H molecules. Active site residues S153 and H180 were changed to alanine before refinement and computation of the electron density in order to locate the side chains unambiguously. Substrate and water molecules shown in stick and sphere models represent their final position after refinement. A-D: Subunits A-D. Click here for file

## References

[B1] Tzipori S, Griffiths JK (1998). Natural history and biology of *Cryptosporidium parvum*. Adv Parasitol.

[B2] Tzipori S, Ward H (2002). Cryptosporidiosis: biology, pathogenesis and disease. Microb Infect.

[B3] Dillingham RA, Lima AA, Guerrant RL (2002). Cryptosporidiosis: epidemiology and impact. Microb Infect.

[B4] Nannini EC, Okhuyesen PC (2002). HIV1 and the gut in the era of highly active antiretroviral therapy. Curr Gastroenterol Rep.

[B5] Coombs GH (1999). Biochemical peculiarities and drug targets in *Cryptosporidium parvum*: lessons from other Coccidian parasites. Parasitol Today.

[B6] Denton H, Brown SM, Roberts CW, Alexander J, McDonald V, Thong KW, Coombs G (1996). Comparison of the phosphofructokinase and pyruvate kinase activities of *Cryptosporidium parvum*, *Eimeria tenella *and *Toxoplasma gondii*. Mol Biochem Parasitol.

[B7] Entrala E, Mascaro C (1997). Glycolytic enzyme activities in *Cryptosporidium parvum *oocysts. FEMS Microbiol Lett.

[B8] Abrahamsen MS, Templeton TJ, Enomoto S, Abrahante JE, Zhu G, Lancto CA, Deng M, Liu C, Widmer G, Tzipori S, Buck GA, Xu P, Bankier AT, Dear PH, Konfortov BA, Spriggs HF, Iyer L, Anantharaman V, Aravind L, Kapur V (2004). Complete genome sequence of the apicomplexan, *Cryptosporidium parvum*. Science.

[B9] Callens M, Hannaert V (1995). The rational design of trypanocidal drugs: selective inhibition of the glyceraldehyde-3-phosphate dehydrogenase in Trypanosomatidae. Ann Trop Med Parasitol.

[B10] Bressi JC, Verlinde CL, Aronov AM, Shaw ML, Shin SS, Nguyen LN, Suresh S, Buckner FS, Van Voorhis WC, Kuntz ID, Hol WG, Gelb MH (2001). Adenosine analogues as selective inhibitors of glyceraldehyde-3-phosphate dehydrogenase of Trypanosomatidae via structure-based drug design. J Med Chem.

[B11] Suresh S, Bressi JC, Kennedy KJ, Verlinde CL, Gelb MH, Hol WG (2001). Conformational changes in *Leishmania mexicana *glyceraldehyde-3-phosphate dehydrogenase induced by designed inhibitors. J Mol Biol.

[B12] Harris JI, Waters M, Boyer PD (1976). Glyceraldehyde-3-phosphate dehydrogenase, in The Enzymes.

[B13] Jenkins JL, Tanner JJ (2006). High resolution structure of human D-glyceraldehyde-3-phosphate dehydrogenase. Acta Crystallogr.

[B14] Castilho MS, Pavão F, Oliva G, Ladame S, Willson M, Périé J (2003). Evidence for the two phosphate binding sites of an analogue of the thioacyl intermediate for the Trypanosoma cruzi glyceraldehyde-3-phosphate dehydrogenase-catalyzed reaction, from its crystal structure. Biochemistry.

[B15] Didierjean C, Corbier C, Fatih M, Favier F, Boschi-Muller S, Branlant G, Aubry A (2003). Crystal structure of two ternary complexes of phosphorylating glyceraldehyde-3-phosphate dehydrogenase from *Bacillus stearothermophilus *with NAD and D-glyceraldehyde 3-phosphate. J Biol Chem.

[B16] Yun M, Park C-G, Kim J-Y, Park H-W (2000). Structural analysis of glyceraldehyde 3-phosphate dehydrogenase from *Escherichia coli*: direct evidence of substrate binding and cofactor-induced conformational changes. Biochemistry.

[B17] Kim H, Feil IK, Verlinde CLMJ, Petra PH, Hol WGJ (1995). Crystal structure of glycosomal glyceraldehyde-3-phosphate dehydrogenase from *Leishmania mexicana*: Implications for structure-based drug design and a new position for the inorganic phosphate binding site. Biochemistry.

[B18] Ladame S, Castilho MS, Silva CHTP, Denier C, Hannaert V, Périé J, Oliva G, Willson M (2003). Crystal structure of *Trypanosoma cruzi *glyceraldehyde-3-phosphate dehydrogenase complexed with an analogue of 1,3-bisphospho-D-glyceric acid. Eur J Biochem.

[B19] Skarżyñski T, Moody PCE, Wonacott AJ (1987). Structure of holo-glyceraldehyde-3-phosphate dehydrogenase from *Bacillus stearothermophilus *at 1.8 Å resolution. J Mol Biol.

[B20] Pavão F, Castilho MS, Pupo MT, Dias RLA, Correa AG, Fernandes JB, da Silva MFGF, Mafezoli J, Vieira PC, Oliva G (2002). Structure of *Trypanosoma cruzi *glycosomal glyceraldehyde-3-phosphate dehydrogenase complexed with chalepin, a natural product inhibitor, at 1.95 Å resolution. FEBS Lett.

[B21] Moras D, Olsen KW, Sabesan MN, Buehner M, Ford GC, Rossmann MG (1975). Studies of asymmetry in the three-dimensional structure of lobster D-glyceraldehyde-3-phosphate dehydrogenase. J Biol Chem.

[B22] Corbier C, Michels S, Wonacott AJ, Branlant G (1994). Characterization of the two anion-recognition sites of glyceraldehyde-3-phosphate dehydrogenase from *Bacillus stearothermophilus *by site-directed mutagenesis and chemical modification. Biochemistry.

[B23] Moniot S, Bruno S, Vonrhein C, Didierjean C, Boschi-Muller S, Vas M, Bricogne G, Branlant G, Mozzarelli A, Corbier C (2008). Trapping of the thioacyl-glyceraldehyde-3-phosphate dehydrogenase intermediate from *Bacillus stearothermophilus*: direct evidence for a flip-flop mechanism. J Biol Chem.

[B24] Senkovich O, Speed H, Grigorian A, Bradley K, Ramarao CS, Lane B, Zhu G, Chattopadhyay D (2005). Crystallization of three key glycolytic enzymes of the opportunistic pathogen *Cryptosporidium parvum*. Biochim Biophys Acta.

[B25] Matthews BW (1968). Solvent content of protein crystals. J Mol Biol.

[B26] Emsley P, Cowtan K (2004). Coot: model-building tools for molecular graphics. Acta Crystallogr.

[B27] Brünger AT, Adams PD, Clore GM, Delano WL, Gros P, Grosse-Kunstleve RW, Jiang J-S, Kuszewski J, Nilges N, Pannu NS, Read RJ, Rice LM, Simonson T, Warren GL (1998). Crystallography and NMR system (CNS): A new software system for macromolecular structure determination. Acta Crystallgr.

[B28] Engh RA, Huber R (1991). Accurate bond and angle parameters for X-ray protein structure refinement. Acta Crystallogr.

[B29] Murshudov GN, Vagin AA, Dodson EJ (1997). Refinement of macromolecular structures by the maximum-likelihood method. Acta Cryst.

[B30] Ostro MJ, Fondy TP (1977). Isolation and characterization of multiple molecular forms of cytosolic NAD-linked glycerol-3-phosphate dehydrogenase from normal and neoplastic rabbit tissues. J Biol Chem.

[B31] Fourrat L, Iddar A, Soukri A (2007). Purification and characterization of cytosolic glyceraldehyde-3-phosphate dehydrogenase from the dromedary camel. Acta Biochim Biophys Sin.

[B32] Marche S, Michels PAM, Opperdoes FR (2000). Comparative study of *Leishmania mexicana *and *Trypanosoma brucei *NAD-dependent glycerol-3-phosphate dehydrogenase. Mol Biochem Parasitol.

[B33] Lambeir A-M, Loiseau AM, Kuntz DA, Vellieux FM, Michels PAM (1991). The cytosolic and glycosomal glyceraldehyde-3-phosphate dehydrogenase from *Trypanosoma brucei*. Kinetic properties and comparison with homologous enzymes. Eur J Biochem.

[B34] Satchell JF, Malby RL, Luo CS, Adisa A, Alpyurek AE, Klonis N, Smith BJ, Tilley L, Colman PM (2005). Structure of glyceraldehyde-3-phosphate dehydrogenase from *Plasmodium falciparum*. Acta Crystallogr.

[B35] Cowan-Jacob SW, Kaufmann M, Anselmo AN, Stark W, Grütter MG (2003). Structure of rabbit-muscle glyceraldehyde-3-phosphate dehydrogenase. Acta Crystallogr.

[B36] Aronov AM, Gelb MH (1998). Synthesis and structure-activity relationships of adenosine analogs as inhibitors of trypanosomal glyceraldehyde-3-phosphate dehydrogenase. Modifications at positions 5' and 8. Bioorg Med Chem Lett.

[B37] Talfournier F, Colloc'h N, Mornon JP, Branlant G (1998). Comparative study of the catalytic domain of phosphorylating glyceraldehyde-3-phosphate dehydrogenases from bacteria and archaea via essential cysteine probes and site-directed mutagenesis. Eur J Biochem.

[B38] Michels S, Rogalska E, Branlant G (1996). Phosphate-binding sites in phosphorylating glyceraldehyde-3-phosphate dehydrogenase from *Bacillus stearothermophilus*. Eur J Biochem.

