# Supplementary figures and images for: An unexpected phosphate binding site in Glyceraldehyde 3-Phosphate Dehydrogenase: Crystal structures of apo, holo and ternary complex of Cryptosporidium parvum enzyme
Source: BMC Struct Biol. 2009 Feb 25;9:9. doi: 10.1186/1472-6807-9-9 (PMC2662861; doi:10.1186/1472-6807-9-9)

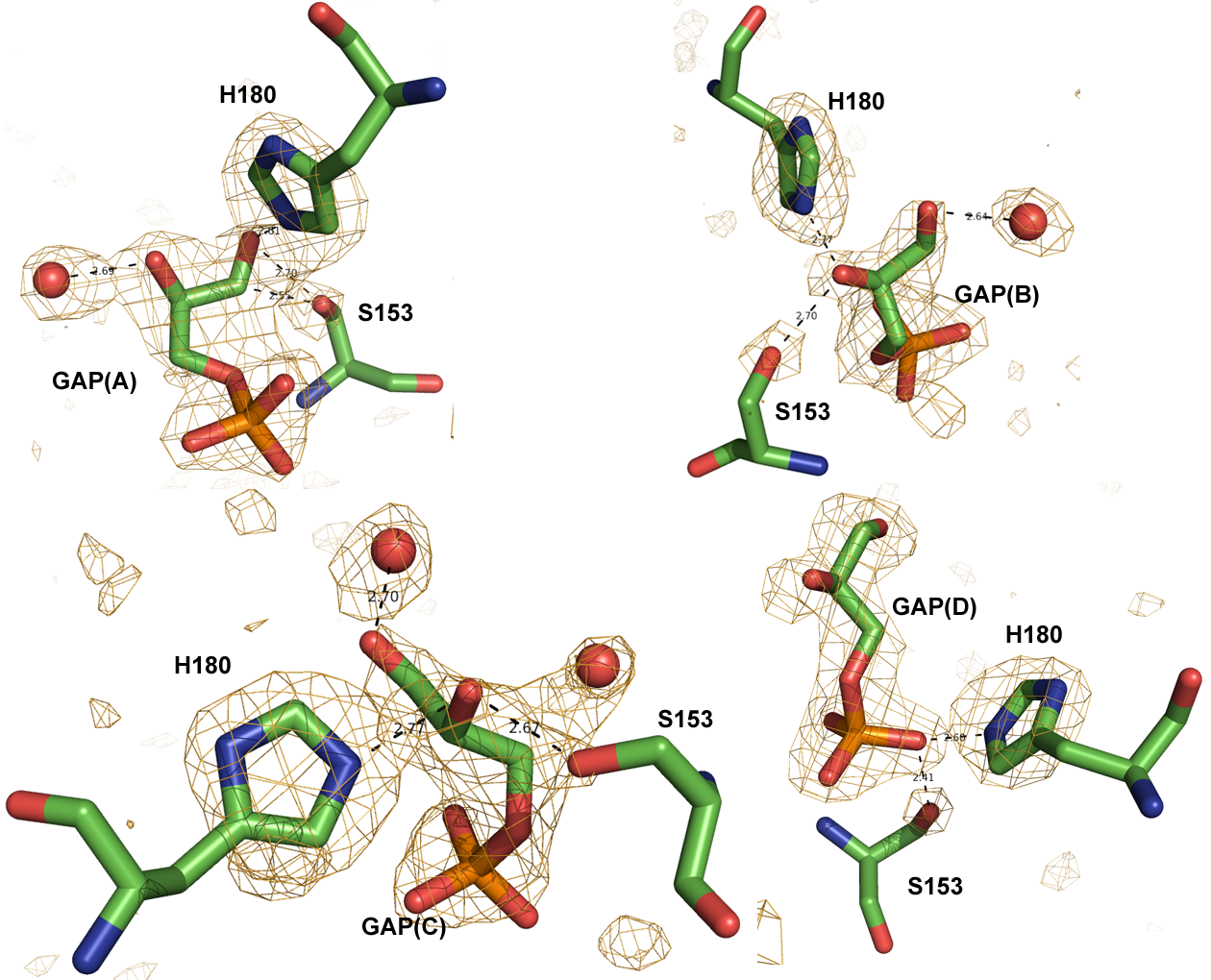

Supplement: Additional file 1 — Substrate binding site in CpGAPDH subunits. Fo-Fc electron density maps contoured at 3.0σ level computed before placement of D-G3H molecules. Active site residues S153 and H180 were changed to alanine before refinement and computation of the electron density in order to locate the side chains unambiguously. Substrate and water molecules shown in stick and sphere models represent their final position after refinement. A-D: Subunits A-D. [file 1472-6807-9-9-S1.tiff]
